# Identification of KRAS mutation in a patient with linear nevus sebaceous syndrome: a case report

**DOI:** 10.1186/s12920-020-00847-1

**Published:** 2020-12-12

**Authors:** Chun Pan, Xiaowei Zhou, Anlan Hong, Fang Fang, Yan Wang

**Affiliations:** Department of Dermatologic Surgery, Hospital for Skin Diseases (Institute of Dermatology), Chinese Academy of Medical Sciences and Peking Union Medical College, Nanjing, 210042 Jiangsu China

**Keywords:** Linear nevus sebaceous syndrome, Schimmelpenning syndrome, Mutation, *KRAS*, *PRKRIR*, *RRP7A*

## Abstract

**Background:**

Linear nevus sebaceous syndrome (LNSS) is a rare genetic disease characterized by large linear sebaceous nevus typically on the face, scalp, or neck. LNSS could be accompanied by multisystem disorders including the central nervous system. Herein, we report gene mutational profile via whole exome sequencing of both lesional and non-lesional skin samples in a LNSS patient.

**Case presentation:**

A 17-year-old girl presented with multisystem abnormalities, including large skin lesions, ocular disorders, abnormal bone development and neurological symptoms. A diagnosis of LNSS was established based on clinical manifestations, histopathological and imaging findings. The skin lesions were resected and no recurrence was noted at the time of drafting this report. Whole exome sequencing of genomic DNA revealed the following 3 mutations in the lesions of the index patient: *KRAS* (c.35G > A, p.G12D), *PRKRIR* (c.A1674T, p.R558S), and *RRP7A* (c. C670T, p.R224W), but no mutation was found in the healthy skin and peripheral blood sample of the index patient, or in the blood samples of her parents and sibling. PCR-mediated Sanger sequencing of DNA derived from lesional skin sample of the index patient verified *KRAS* mutation, but not *PRKRIR* (c.A1674T, p.R558S) and *RRP7A* (c. C670T, p.R224W). None of the 3 mutations was found in Sanger sequencing in skin lesions of 60 other cases of nevus sebaceous patients.

**Conclusions:**

Our findings show the relevance of KRAS mutation to LNSS, providing new clues in understanding related genetic heterogeneity which could aid genetic counselling for LNSS patients.

## Background

Linear nevus sebaceus syndrome (LNSS), also known as Schimmelpenning Syndrome, is a rare multisystem disorder with a spectrum of anomalies, including central nervous system, ocular, skeletal and cardiovascular defects [1]. Nevus sebaceous, a hallmark of LNSS, occurs in 0.1–0.3% newborns [2–4]. Clinically, LNSS is characterized by hairless, yellow–orange plaques of varying sizes and shapes, and is most frequently located in areas with abundant sebaceous glands. Epidermal and skin adnexal changes, including acanthosis, sebaceous gland hyperplasia and ectopic apocrine glands, are also common [5, 6].

Familial cases of both LNSS and nevus sebaceous have been reported. Para-dominant inheritance was initially thought to be responsible for the familial aggregation, but has been disputed [7]. Some scholars speculate that both LNSS and nevus sebaceous result from genetic mosaicism during embryogenesis, which is limited to the skin in nevus sebaceous but involves other organs in LNSS [8]. The extent of mutations, as well as body site-specific embryologic patterns and environmental factors, may contribute to phenotypic difference [4]. The elucidation of the genetic basis of nevus sebaceous and its associated syndromes is crucial for understanding genotype–phenotype correlations and genetic mosaicism [9].

In the current study, we examined the gene mutational profile using whole‐exome sequencing (WES) of both lesional and non-lesional skin samples in a patient with LNSS. Sanger sequencing was performed in 60 other cases of nevus sebaceous to verify the findings.

## Case presentation

A 17-year-old girl presented on January 2, 2014 with multiple skin lesions. The lesions were reported to be present at birth on the right scalp, face, neck and trunk. *Café-au-lait* patches appeared in both arms and gradually coalesced. The lesions on the head and neck were characterized by warty protuberances whereas the lesions on the right face were thickened, with new protruding nodules.

Past history included febrile convulsion, right lateral canthus soft tissue tumor at 12, worsening exotropia of the right eye, and social communication disorder. Family history was unremarkable.

**Fig. 1:**
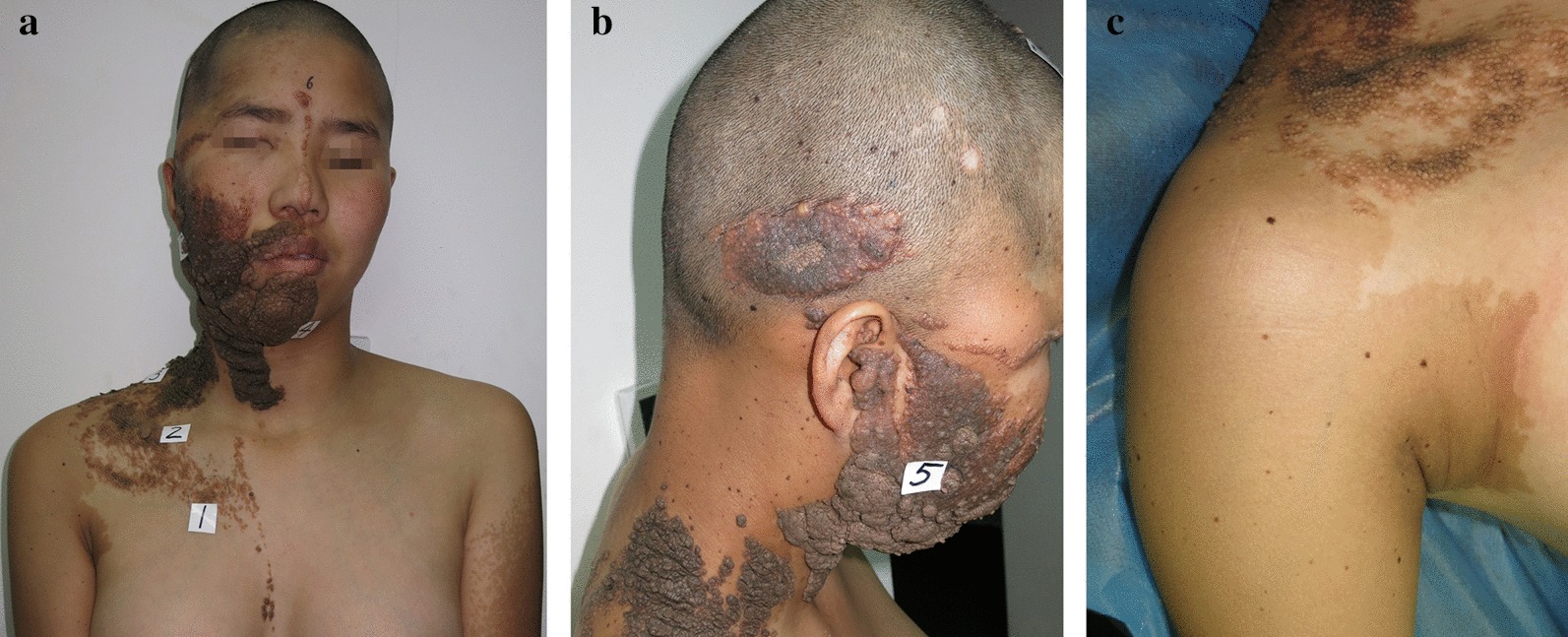
**a**, **b** Nevus sebaceous is seen on the right scalp, ear, oral and nasal cavity, neck, and chest. Strabismus and ptosis are noticed. **c** Generalized nevus spilus with speckled lentiginous nevus is seen on the shoulder and upper limb

**Fig. 2:**
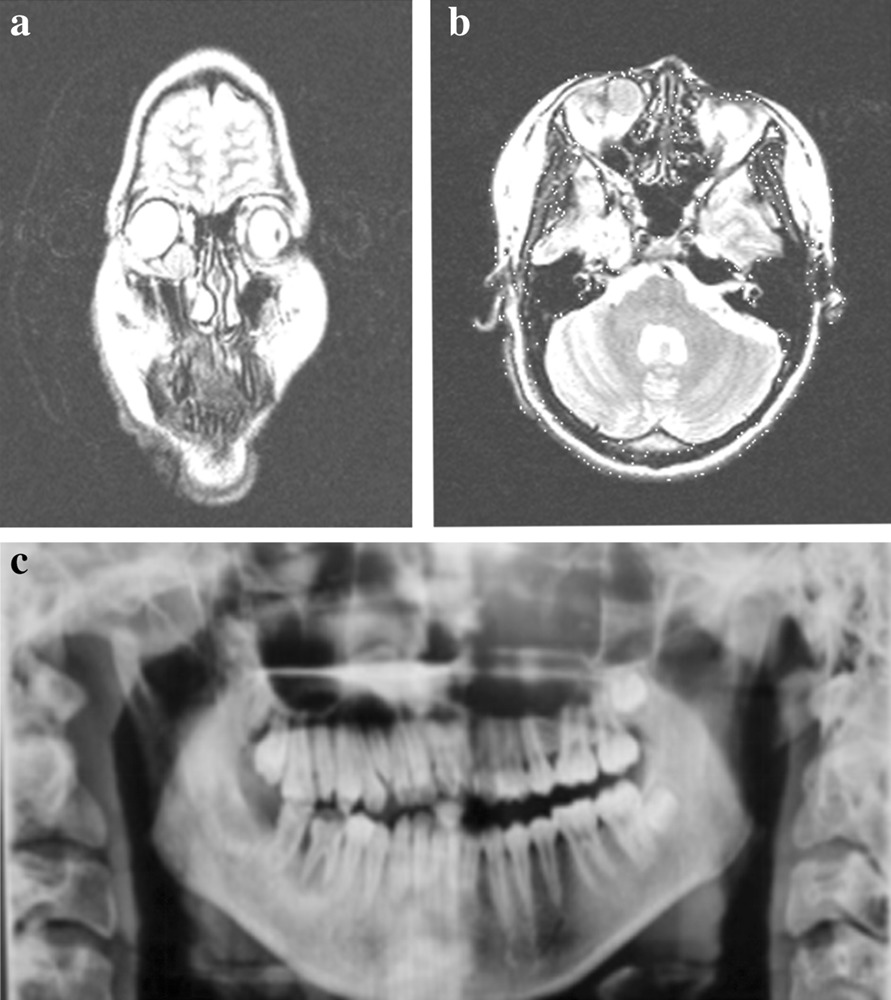
**a**, **b** Plain orbital MRI scan reveals an 18-mm diameter abnormal intensity signals in the right lacrimal sac. T1WI shows hyperintensity while T2WI shows hypointensity. Partial destruction of the right orbital bone and ethmoid bone is noticed. **c** Orthopantomography shows poor development of the right upper and lower jaw bones as well as alveolar bones

**Fig. 3:**
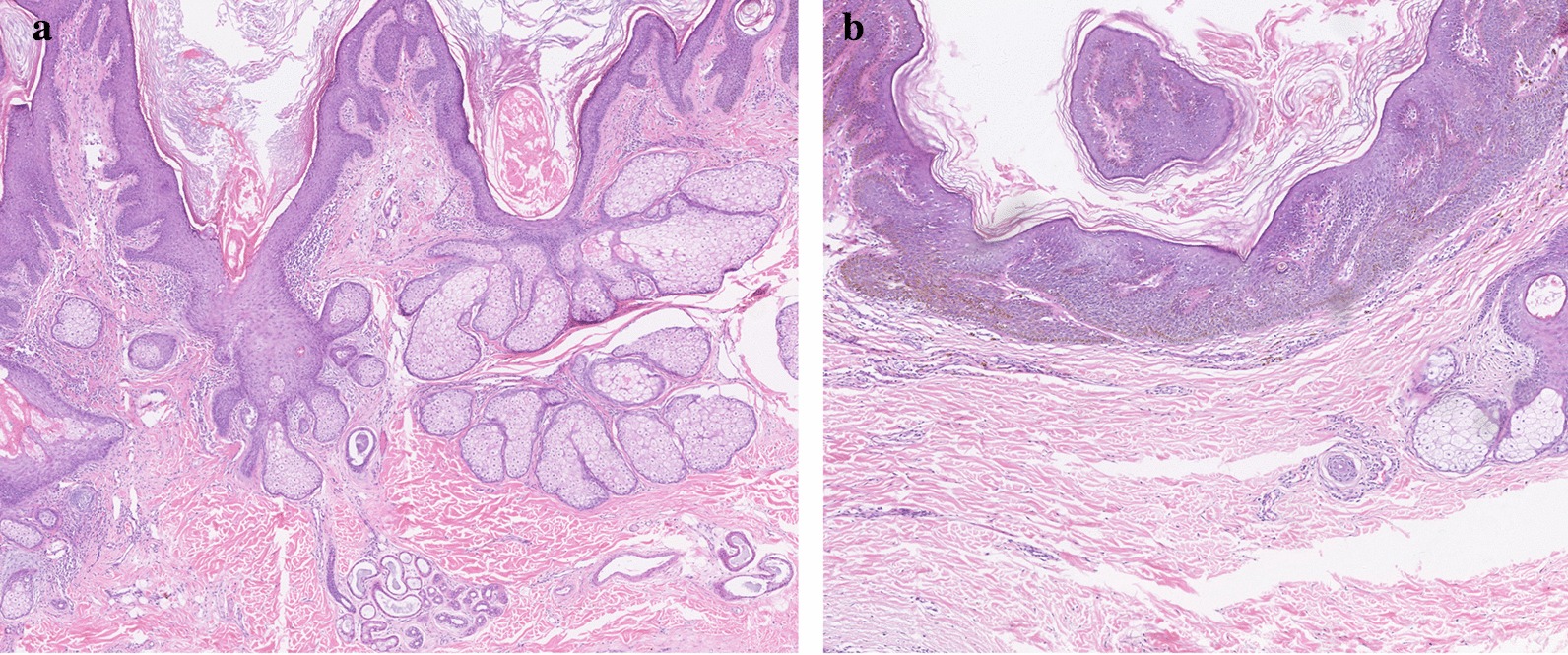
**a** H&E staining of skin lesions in the head and face reveals epidermal hyperkeratosis, acanthosis, and papillomatous hyperplasia in the epidermis, numerous mature/nearly mature sebaceous glands in the dermis, and sparse ectopic apocrine sweat glands. **b** Histopathological examination of skin lesions on the chest reveals an increased number of nevus cells in the basal layer and numerous mature or immature sebaceous glands in the dermis. Magnification × 50

Physical examination revealed large nevus sebaceous on the scalp, oral and nasal cavity, neck, and chest, and generalized nevus spilus on the arm, shoulder, and back (Fig. 1). Strabismus and ptosis were noted. Optical coherence tomography scan showed reduced diameter of the right optic disc. Plain orbital MRI scan demonstrated abnormal intensity signals, 18 mm in diameter, in the right lacrimal sac. T1WI showed hyperintensities whereas T2WI revealed hypointensities (Fig. 2a, b). The right orbital and ethmoid bone were partially destroyed. Orthopantomography showed poor development of the right upper and lower jaw bones, as well as alveolar bones (Fig. 2c). H&E staining of skin lesions in the head and face revealed epidermal hyperkeratosis, acanthosis, papillomatous hyperplasia in the epidermis, numerous mature or immature sebaceous glands in the dermis, and sparse ectopic apocrine sweat glands (Fig. 3a). Lesions on the chest showed an increased number of nevus cells in the basal layer and numerous mature or immature sebaceous glands in the dermis (Fig. 3b). A diagnosis of LNSS was made.

The skin lesions was resected on January 16, 2014. Post-operative recovery was unremarkable except for mild scar hyperplasia at the surgery sites. At the time of drafting this manuscript, no recurrence was reported.

## Mutational profile

**Fig. 4:**
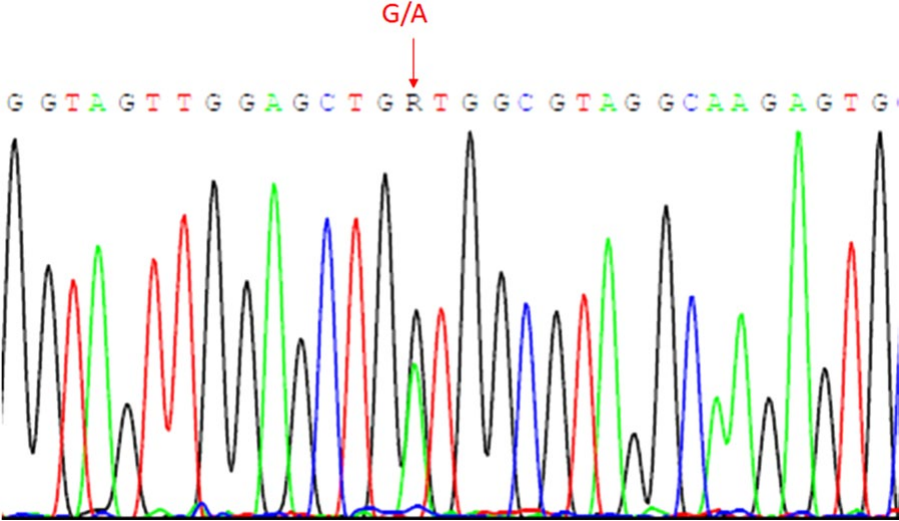
PCR-mediated Sanger sequencing in the lesional tissue of the index patient detected KRAS (NM004985, NM_033360)

WES analysis of genomic DNA was conducted to examine gene mutations in lesional and non-lesional samples of the index patient, peripheral blood samples from the patient, her parents and sibling. Sanger sequencing was conducted to examine lesional samples of the index patient and 60 additional patients with nevus sebaceous (Additional file 1: Methods). Among the 89 genes and 172 loci of somatic mutations (list available upon request), we identified three candidate loci: exon 2 of *KRAS* (NM004985, NM_033360) (c. 35G > A, p.G12D, allele frequency 28%, sequencing depth 100 ×), exon 5 of *PRKRIR* (NM004705) (c.A1674T,p.R558S, allele frequency 7.7%, sequencing depth 100 ×), and exon 6 of *RRP7A* (NM_015703) (c. C 670T, p.R224W, allele frequency 4.8%, sequencing depth 100 ×) in the lesions from the index patient (Table 1). No mutations were found in *BRAF* and *HRAS*. Deep sequencing with peripheral blood samples of the family did not reveal meaningful mutations. PCR-mediated Sanger sequencing have found *KRAS* (NM004985, NM_033360) (Fig. 4), but not *PRKRIR* (NM004705) or *RRP7A* (NM_015703) in the lesional sample of the index patient. None of the 3 mutations was found in Sanger sequencing in the lesions of 60 other patients with nevus sebaceous. Peripheral blood or healthy tissue were not used in Sanger sequencing.

**Table 1:** Mutational profile of the LNSS by whole exome sequencing

Gene name	CHROM	POS	Gene (Transcript ID)
*PRKRIR*	11	76062520	NM_004705
*KRAS*	12	25398284	NM_004985, NM_033360
*RRP7A*	22	42910199	NM_015703

## Discussion and conclusions

We previously reported a rare case of LNSS with multisystem abnormalities [1]. In the current study, WES and Sanger sequencing of lesional and non-lesional genomic DNA samples demonstrated postzygotic mosaicism mutations of *KRAS*, *PRKRIR* and *RRP7A* in LNSS lesions.

It is well accepted that postzygotic *RAS* mosaicism mutations cause LNSS and nevus sebaceous. The formation of cutaneous hamartomas and multiorgan abnormalities is believed to be the consequence of downstream signaling pathway activation. RAS isoforms play a crucial role in regulating cell survival, proliferation and differentiation [7–9]. Germline mutations in RAS and other members of the MAPK pathway (e.g., *KRAS*, *HRAS* and *NRAS*) may cause rare inherited disorders known as “RASopathies”, including LNSS and nevus sebaceous [7, 10]. In a study that included 2 LNSS patients and 63 patients with nevus sebaceous, Groesser et al. identified *HRAS* mutations (mostly c.37G > C) in 95% of the cases, and *KRAS* mutations in the remaining 5% of the cases [8]. Sun et al. [9] found *HRAS* (c.37G >, c.35G > A) or *KRAS* (c.35G > A) mutations, in 89% of the specimens from a LNSS patient and 31 patients with nevus sebaceous. Apart from postzygotic mosaicism mutations of *HRAS* and *KRAS*, postzygotic *NRAS* mutation has been reported in a case of LNSS [1].

Mutations in codon 12 of *KRAS* result in constitutive activation. Somatic *KRAS* mutations are frequently detected in lung, colorectal, and pancreatic cancers. Based on the Catalogue of Somatic Mutations in Cancer (COSMIC) database, *KRAS* c.35G > A is the most frequent *KRAS* mutation in human cancers [8]. In mice, *KRAS* c.35G > A is not tolerated during embryonic development. Similarly, this mutation has not been observed in human germline cells [8]. A shared somatic mosaic *KRAS* mutation (c.35G > A) has been reported in the cutaneous lesions of 4 cases of LNSS [2, 11–13]. A recent study proposed that the mutation could account for most cases of LNSS [10].

Postzygotic *KRAS* mosaicism mutation has been proposed by previous studies [3, 8]. Observed discordance in monozygotic twins also supports a model of postzygotic somatic mutation in LNSS [14, 15]. In the current study, we identified a *KRAS* mutation (c.35G > A; p.G12D) in the skin lesions of the index patient, but not in her peripheral blood sample and un-lesional skin. Such a finding is consistent with postzygotic somatic mutation. *PRKRIR* (c.A1674T) and *RRP7A* (c. C 670T) mutations were not verified in Sanger sequencing in the lesional samples of index patient and 60 other patients with nevus sebaceous, indicating that *PRKRIR* and *RRP7A* mutations may not be responsible for LNSS.

The epidermal nevus syndromes represent a group of distinct disorders that can be distinguished by the type of associated epidermal nevus and the presence or absence of heritability. Syndromes characterized by organoid epidermal nevi include LNSS, phacomatosis pigmentokeratotica and other syndromes [16]. Nevus sebaceous, the hallmark of LNSS, is commonly found in the scalp (59.3%) and face (32.6%) and only occasionally in the neck and trunk [17]. The index patient had diffuse sebaceous nevi covering the right half of her body from head to chest. Additionally, large areas of nevus spilus with speckled lentiginous nevus were present on the posterior side of her arms. Thus, differential diagnosis must be made to exclude phakomatosis pigmentokeratotica [18, 19], but we did not perform WES using nevus spilus tissue.

Approximately 7% of nevus sebaceous cases exhibit extracutaneous manifestations, most commonly in the central nervous system [20, 21]. LNSS is also often complicated with ocular abnormalities, including colobomas and choristomas [3] and occasionally strabismus and esotropia [22, 23]. The lacrimal passage and puncta develop at 6–7 weeks during embryogenesis [24, 25]. We believe the right lower lacrimal punctum atresia in the index patient is congenital, but do not have conclusive evidence. The loss of orbital inferior wall bone is usually caused by congenital bone dysplasia [26], whereas ptosis is caused by complete or partial functional loss of the levator palpebral muscle (oculomotor innervation) and Müller muscle (cervical sympathetic nerve innervation) [27, 28]. Limited movement and ptosis of the right eye in the index patient may be caused by compression by the dacryocyst against the inferior oblique muscle and the medial rectus muscle, but the possibility of congenital abnormal development of the oculomotor nerve could not be ruled out.

In conclusion, we identified *KRAS* mutation and previously unreported *PRKRIR* and *RRP7A* mutations in a patient with LNSS. Comparison of the mutation profile with healthy family member and patients with nevus sebaceous implicated *KRAS* mutation to LNSS and not in nevus sebaceous.

## Supplementary Information

**Additional file 1:** Supplementary methods.

## Data Availability

The data shown in this report are not publicly available because it contains information that could compromise patient privacy. Data are available from the corresponding author upon reasonable request. The datasets generated and/or analyzed during the current study are available in the human reference genome (GRCh37/hg19, https://www.ncbi.nlm.nih.gov/genome/guide/human/), NM004985, NM_033360, NM004705, NM_015703 (https://www.ncbi.nlm.nih.gov/nuccore).

## Abbreviations

LNSS: Linear nevus sebaceous syndrome
PCR: Polymerase chain reaction
MAPK: Mitogen-activated protein kinase
COSMIC: The Catalogue of Somatic Mutations in Cancer database
