# Supplementary material for: Identification of KRAS mutation in a patient with linear nevus sebaceous syndrome: a case report
Source: BMC Med Genomics. 2020 Dec 12;13:188. doi: 10.1186/s12920-020-00847-1 (PMC7733249; doi:10.1186/s12920-020-00847-1)
Supplement: Supplementary file 1 — Additional file 1: Supplementary methods. [file 12920_2020_847_MOESM1_ESM.docx]

**Supplementary Methods**

Genomic DNA was extracted from the lesional epidermis/dermis from post aurem and the contralateral non-lesional epidermis of our LNSS patient. Peripheral blood samples were collected from the patient and her unaffected parents and younger brother. Genomic DNA was also extracted from the skin and blood samples using the protocols recommended by the manufacturer (Qiagen DNA kit, Germany). Next, whole-exome sequencing was done using Agilent SureSelect Human All Exon v6 Kit (Agilent, Santa Clara, CA, U.S.A.) and an Illumina platform (Illumina, San Diego, CA, U.S.A.) according to the manufacturers’ protocols. Sequencing data were aligned to GRCh37/hg19 using the Burrows-Wheeler Aligner software (v.0.7.8) and polymerase chain reaction (PCR) duplicates were removed with the Samtools software (v.1.0). ANNOVAR (v. 2013Aug23) was utilized to annotate the detected variations. Variants were filtered against the databases of dbSNP (<https://www-ncbi-nlm-nih-gov-443.webvpn.cams.cn/SNP/>), the 1000 Genomes Project (<http://www.1000genomes.org/>), ExAC database (<http://exac.hms.harvard.edu/>), HGMD (<http://www.hgmd.cf.ac.uk/ac/index.php>) and OMIM (<https://www-ncbi-nlm-nih-gov-443.webvpn.cams.cn/omim/>). Then, we performed Sanger sequencing and data analysis using the DNA sample extracted from the lesional tissue of the LNSS patient and 60 other nevus sebaceous patients. Primers were designed based on the filtered variants. The PCR amplified DNA samples were sequenced on an ABI 3730 sequencer and the results were analyzed with applied BioSystems sequencer software.
